# Volunteers in the Smart City: Comparison of Contribution Strategies on Human-Centered Measures

**DOI:** 10.3390/s18113707

**Published:** 2018-10-31

**Authors:** Stefano Bennati, Ivana Dusparic, Rhythima Shinde, Catholijn M. Jonker

**Affiliations:** 1 Chair of Computational Social Science, ETH Zürich, Clausiusstrasse 50, 8092 Zürich, Switzerland; 2 School of Computer Science and Statistics, Trinity College Dublin, Dublin 2, Ireland; duspari@scss.tcd.ie; 3 Interactive Intelligence Group, TU Delft, Mekelweg 4, 2628 Delft, The Netherlands; shinde@ifu.baug.ethz.ch (R.S.); C.M.Jonker@tudelft.nl (C.M.J.); 4 LIACS, Leiden University, Niels-Bohr-Weg 1, 2333 CA Leiden, The Netherlands

**Keywords:** participatory sensing, smart cities, public good, privacy, fairness

## Abstract

Provision of smart city services often relies on users contribution, e.g., of data, which can be costly for the users in terms of privacy. Privacy risks, as well as unfair distribution of benefits to the users, should be minimized as they undermine user participation, which is crucial for the success of smart city applications. This paper investigates privacy, fairness, and social welfare in smart city applications by means of computer simulations grounded on real-world data, i.e., smart meter readings and participatory sensing. We generalize the use of public good theory as a model for resource management in smart city applications, by proposing a design principle that is applicable across application scenarios, where provision of a service depends on user contributions. We verify its applicability by showing its implementation in two scenarios: smart grid and traffic congestion information system. Following this design principle, we evaluate different classes of algorithms for resource management, with respect to human-centered measures, i.e., privacy, fairness and social welfare, and identify algorithm-specific trade-offs that are scenario independent. These results could be of interest to smart city application designers to choose a suitable algorithm given a scenario-specific set of requirements, and to users to choose a service based on an algorithm that matches their privacy preferences.

## 1. Introduction

Recent years have seen a substantial increase in active user participation in the smart city, and, with it, an increase in the resources contributed by the citizens. Sensor data is one type of user-contributed resource that is at the base of many smart city applications [1]. Collecting data in a smart city allows the applications to predict the needs of the citizens [2], thus enabling the creation of more advanced and more efficient services with a high potential for innovation [3].

User participation is crucial for the success of some smart city applications, but it entails costs that disincentivize users. For example, transmitting privacy-sensitive data in a participatory sensing scenario increases the risk of disclosure and misuse of private information, e.g., discrimination. Similarly, increasing energy availability in the smart grid scenario, e.g., by postponing the use of appliances, comes with a risk of disproportionately low access to the resource and unfair treatment [4]. In order for the smart city applications to be successful, these costs and risks must be reduced.

Examples of existing solutions for reducing contribution costs are privacy-enhancing technologies [5] that reduce the risks associated with disclosing information [6], and fair resource-management technologies [7], which improve the perceived fairness of the system. However, comparing different implementations of smart city algorithms is difficult because they are tied to specific assumptions about the scenario and the resource.

The first contribution of this paper is to introduce a design principle that enables such a comparison by means of a scenario-independent model of user contributions to a smart city application, which is based on the theory of public goods and voluntary contribution games [8]. This principle is based on the concept of contribution strategy: the orchestration of demand and supply of some common resource, i.e., the smart city service, is driven by independent contributions of individuals in a population. This approach is independent of the characteristics of the resource; hence it can be combined with existing mechanisms that work at the content level, e.g., privacy-preserving algorithms. This also makes data sharing across multiple smart city applications easier, thanks to the uniform way of dealing with different kinds of data.

Following this design principle, a simulation framework is developed (available on Github at [9]) with the goal of understanding user participation in smart city services by addressing the research question: how do different contribution strategies compare in terms of privacy, fairness and social welfare?

The second contribution of this paper is to verify the applicability of the design principle in two different examples of smart city application scenarios, i.e., traffic congestion information and charging of electric vehicles, using real-world datasets. Similar validation in different scenarios can provide useful insights for the design of other resource-based smart city applications.

The third contribution is to compare different contribution strategies—centralized optimization and two flavors of localized learning, with and without contextual awareness—in terms of trade-offs between system efficiency, user privacy or resource distribution fairness, and to identify scenario-independent trade-offs that characterize a specific algorithm.

Specifically, localized solutions, which work only on local data, are found to deliver higher privacy and equality than a centralized solution. Centralized optimization offers instead higher efficiency thanks to the global knowledge of data. Localized algorithms with and without contextual knowledge are evaluated, and those considering the current context in the decision-making are found to deliver higher fairness.

This work can be of interest to designers of smart city applications and services that look for a guideline for the choice of algorithm, given specific scenario priorities in terms of different measures. Another target audience is that of citizens that are concerned with the risk of abuse of their data, and particularly privacy-aware prosumers. The results help quantify different service implementations along measures such as privacy and fairness; thus help the citizens choose a service provider that best matches their preferences, reducing the concerns about the system and fostering user participation.

The rest of the paper is organized as follows: Section 2 introduces the proposed design principle and describes how it can be applied to two smart city scenarios, Section 3 describes the contribution strategies and the measures used to evaluate them, Section 4 describes and discusses the main results obtained from simulating voluntary contributions in different scenarios, Section 5 situates the work in the literature, and Section 6 concludes the paper discussing possible avenues for future work and giving general recommendations about the choice of contribution strategies.

## 2. Model

This section introduces the design principle, based on the theory of public goods and Voluntary Contribution Games [8], and it describes how it can be applied to the smart city scenarios of participatory sensing, with the example of traffic congestion [10], and smart grids, with the example of electric vehicle charging [11]. Voluntary contribution games involve the need of coordination between users to provide some public good, e.g., financing a playground with private donations. The contribution of this paper is to present Voluntary contribution games as a design principle for modeling generic smart city services, and not limited to modeling only a specific scenario, e.g., smart grids [12]. The rest of this section presents the details of our proposed design principle. Table 1 illustrates the notation of the Voluntary contribution games model; symbols are listed in order of appearance.

V={1,…,n} is the set of users and *S* is a service provider. In each round t≤T, each user produces a resource $r_{i}$ with value $v_{i}$. Users perform an action $a_{i}\in A=\left\{C,D\right\}$: *C* corresponds to contributing the resource and *D* to opt out from contribution. The set of contributors is denoted as $A_{+}=\left\{i\in V:a_{i}=C\right\}$.

Contribution is costly; thus, contributors pay a cost $c_{i}$ that depends on the characteristics of resource and communication medium. Contribution might also entail a privacy cost $p_{i}$, which models the risks of revealing private information to third parties.

The service provider determines the service quality $q=f\left(A_{+}\right)=\sum_{i\in A_{+}}v_{i}$ based on all contributions received. A quality requirement τ, either global or per user, is generated at every timestep. Not all users are required to contribute in order to satisfy the requirement $\tau \leq \sum_{i}v_{i}$. A round is successful, i.e., the service can be provided, if the quality is higher than the threshold: the success function is defined as S(q,τ)={Gifq≥τelseB}. Each user gets the same positive payoff G=G(τ,q) from accessing the service, including those who did not contribute. If the quality threshold is not met, the service cannot be provided and every agent receives a large negative payoff B(τ,q)>0. Given that payoffs are distributed equally, there is no incentive for users to contribute unilaterally: the public goods theory predicts the existence of an equilibrium where nobody contributes.

The individual goal of the users is to maximize their individual utility over *T* rounds (see Table 2):

$$U_{i}=\left\{\begin{array}{cc} G(\tau ,q) & \;\mathrm{if}\;q\geq \tau \\ -B(\tau ,q) & \;\mathrm{if}\;q<\tau \end{array}\right.-\left\{\begin{array}{cc} c_{i} & \;\mathrm{if}\;a_{i}=C,\\ 0 & \;\mathrm{otherwise}. \end{array}\right.$$

The social goal, i.e., the goal of the service provider, is to maximize the total quality of the service over *T* rounds $Q=\max\sum_{t=1}^{T}f\left(A_{+}\right)$.

The model relies on the following assumptions:

- Users contribute their resources to a central entity, which uses them to provide a service.
- Contributions cannot be doctored, e.g., to reduce the cost.
- Users are not allowed to communicate with each other; this is generally the case for simple devices such as smart meters.
- The system implements a privacy-preserving resource-management algorithm that optimizes the use of the resource.

### 2.1. Implementation of the Model in Real-Life Scenarios

This section discuses the generality of the proposed model by describing how real-life data-based smart city applications can be modelled using our proposed design principle. We introduce smart grid (electric vehicle charging) and traffic congestion information (based on data contributed by participatory sensing) scenarios. We describe the data used by the model and illustrate examples of a public good, contribution, cost and value in each scenario.

#### 2.1.1. Smart Grid: EV Charging

In the smart grid scenario, we assume a neighborhood composed of households connected to each other via the infrastructure of the smart grid. Households consume a variable quantity of energy, depending on the appliances in use, and produce a variable quantity of renewable energy, depending on weather conditions. Electric vehicles (EVs), associated with households connected to the smart grid, are required to be periodically recharged. Demand-side management mechanism [13] is required to schedule and postpone appropriately EV charging, based on current demand and renewable energy production, i.e., energy availability.

The *public good* consists of the total energy surplus σ=π−β, which is available to all households for charging the EVs. Its *value* is computed from the current production of renewable energy $\pi =\sum_{i}\pi_{i}$, and the current network load $\beta =\sum_{i}\beta_{i}$[14]. We assume that the energy surplus is lower than the total demand, i.e., at any given time π<β; hence, a black-out is inevitable if all households decide to use the surplus simultaneously. *Contribution* to the public good is then defined as opting out from consumption, i.e., renouncing to charge the EV of a charge $v_{i}$ that might depend on contextual variables such as the current charge level, the availability of a charging station, and the current energy surplus. It is assumed that the values are privacy-sensitive as they might be used to infer habits, e.g., the work schedule, of the owners. Contribution entails a comfort cost that is assumed to be proportional to the corresponding value, as the utility of an EV depends on it being charged. *Values* $v_{i}$, which corresponds to the charge that the EV can accumulate during a time period, are generated uniformly at random between 1 and n2, while the corresponding *costs* $c_{i}$ are determined by sampling a normal distribution centered around the value $v_{i}$. The threshold τ is dynamically computed at every timestep from the dataset to be inversely proportional to the difference between production and consumption: $\tau =1-(\pi_{i}-\beta_{i})$.

The baseload level β—non reschedulable load for each household—determines the aggregated consumption of the network and is obtained from Irish Smart Meter trial data [15]. On a single household level, on average, it ranges from 0.8 kW, during the night, to 3 kW, at evening peak time. The energy production level π is based on Irish wind production data, obtained from Irish electricity grid operator EirGrid [16], which represents an estimate of the total electrical output of all wind farms on the system. Individual contribution values and costs are randomly sampled from a uniform distribution that ranges from 1 to a maximum value that is determined by the simulations’ parameters.

#### 2.1.2. Participatory Sensing: Traffic Congestion Information

In this scenario, we assume a service provider which optimizes the congestion of the whole road network [17] and provide live traffic information to the users. Traffic statistics are considered a *public good* as their creation requires that enough users *contribute* their mobility traces to the service provider [18]. The value $v_{i}$ of individual measurements reflects the novelty of the information, which might depend on the actions of other users [19], e.g., duplicated information due to local correlation in measurements. In this specific case, measurements cannot be linked with one another as the dataset lacks GPS coordinates; therefore, the *value* of novelty is approximated with the change of speed, as a sudden change in speed is considered more informative than keeping a constant speed.

In this scenario, contributing entails a privacy *cost* that comes from revealing privacy-sensitive information such as an individual’s location [20] or destination, as this information can reveal users’ habits, even if the locations are obfuscated [21,22]. Another source of private information is the travel speed, as it might reveal violation of the road laws. Depending on the contribution strategy, private information might be disclosed even by non-contributors, e.g., computing the optimal allocation of contributors might require all users to reveal their location.

Real mobility traces of cars are provided by the 2011 Atlanta Regional Travel Survey by the U.S. Department of Energy, National Renewable Energy Laboratory (NREL) [23], containing travel speed of 25,797 participants, encoded second-by-second GPS information (speed) over the course of three days. Trips with less than 1000 data points are removed from the dataset, which results in a total of 663 people in 377 households. The values and cost are generated from the data and rescaled within the range from 1 to a maximum value that is determined by the simulation’s parameters. The distribution of frequencies of value/cost pairs in the NREL dataset is shown in Figure A3 in the Appendix.

**Figure 1:**
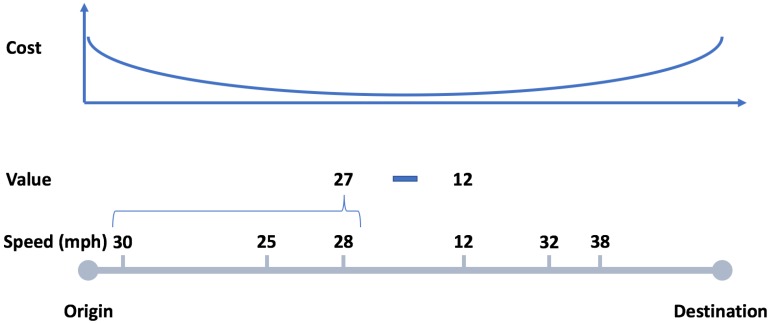
Definition of costs and values in the traffic data. Costs are defined as the distance from the source and the destination, with the assumption that knowing these location conveys privacy-sensitive information. The costs are maximal at the source and at the destination, and decrease in between. Values are defined as the difference between the current speed and the average past speed, with the assumption that a sudden change of speed conveys information about traffic congestion.

Contributing data comes at a transmission cost $c_{i}$—that might depend on the characteristics of the message, e.g., size, and of the medium, e.g., congestion, power consumption—but also, most relevantly to our scenario, privacy cost $p_{i}$—that might depend on disclosing private information, such as location and speed [21]. Therefore, *costs* are determined as the distance to the only points of interests known in the data: the origin and destination of a trip. The cost $c_{i}$ is the highest at either the source or the destination and decreases as the distance from these points increases (see Figure 1). The *public good* is created if the sum of individual contributions is higher than a certain threshold τ, which is set to 80% of the size of the population, which means that the service is successfully generated if at least 80% of the users contribute a value of 1.

## 3. Evaluation of Contribution Strategies

Following the implementation of the two smart-city scenarios which rely on user data contributions described in the previous section, we now present three commonly used algorithms in smart city applications, which we implement in both of these scenarios, in order to evaluate them with respect to human-centred measures. We first describe in detail the implementation of the algorithms on which contribution strategies are based, and then describe the measures on which the contribution strategies are evaluated.

Broadly, algorithms for optimisation in smart cities can be classified into centralized and distributed algorithms. Centralized contribution strategies translate in the real world into optimization algorithms that are implemented by a service provider, e.g., load balancing in smart grids, where contributions are decided at the central level and users have no decisional power. In contrast, distributed strategies give the choice to the users allowing them to act independently of others and reflecting their personal privacy concerns. These distributed strategies model human decision-making by evaluating the trade-off between the benefits from accessing the service and an estimation of the privacy cost for the user. In reality, human decision is more complex than this, e.g., the value of privacy expressed by people does not necessarily reflect in their actions, the so-called privacy paradox [24]. The purpose of these contribution strategies is not to model accurately human behavior, but instead to evaluate whether distributed decision-making, possibly performed by the users themselves, that optimizes for the individual utility would compare against centralized decision making that optimizes for the global utility.

### 3.1. Algorithms

This section describes the contribution strategy algorithms chosen for the analysis in our framework. The criteria for the choice of algorithms is their diffusion and application to smart city scenarios. Enough background and details on the algorithms are given to provide the basis for understanding the specificity of our implementation, which are then described together with their parameters and the evaluation metrics. The contribution of this paper is to compare these algorithms on several smart city application scenarios and verify that trade-offs between evaluation measures depend on the algorithms and are independent of the application scenario. Our algorithm choice favored well-established and general-purpose algorithms as opposed to algorithms with state-of-the-art efficiency, as the goal is to highlight trade-offs between measures over different scenarios. The review and comparison of scenario-specific algorithms are not aligned with the goals of this paper and are therefore out of scope.

#### 3.1.1. Centralized Algorithms: Optimisation

Centralized or top-down algorithms rely on a central optimizer that satisfies the public good while minimizing the cost of contribution. This problem can be modeled as the well-known Knapsack problem: minimize $\sum_{i=1}^{n}c_{i}*a_{i}$ subject to $\sum_{i=1}^{n}v_{i}*a_{i}>\tau$, where the weight of items is given by the contribution value and the value of each item is defined as the inverse of the cost. We chose a customized “fully polynomial time approximation scheme” (FPTAS) that reaches the knapsack constraints from above, instead of from below, such that the threshold can be met.

#### 3.1.2. Localized Algorithms

Decentralized algorithms distribute decision-making at the local level and allow communication between agents for coordination [25] or learning [26]. In this paper, we focus on *localized* algorithms, a type of decentralized algorithms that operate only on local knowledge, without assuming the availability of specialized hardware for communication [27].

##### Aspiration Learning

Aspiration learning is a learning algorithm that is specifically tailored for coordination games [28]. Agents contribution does not consider the current context, e.g., current value or cost; it is instead based on the agent’s *aspiration value*, which determines how satisfied the agent is with the status quo, given its previous experiences in the game.

At every turn, the agent updates its aspiration level $\rho_{i}\left(t\right)$ and chooses an action α(t). The action for the current turn will be the same action executed in the past turn if the current reward exceeds the aspiration level; otherwise, a random action will be selected with probability

$$p_{i}\left(t\right)=1-\max\left(h,1+c*\left[u_{i}\left(\alpha \left(t\right)\right)-\rho_{i}\left(t\right)\right]\right),$$

where $u_{i}\left(\alpha \left(t\right)\right)$ is the reward obtained for executing action α(t). The aspiration level is updated according to the following formula:

$$\rho_{i}\left(t+1\right)=\rho_{i}\left(t\right)+\epsilon \left[u_{i}\left(\alpha \left(t\right)\right)-\rho_{i}\left(t\right)\right]+r_{i}\left(t\right),$$

where $r_{i}\left(t\right)$ is a noise component and the value of $\rho_{i}\left(t+1\right)$ is bound between $\bar{\rho }$ and $\underline{\rho }$.

The algorithm is implemented following the details contained in the original paper, with the following choice of parameters: $\lambda =10^{-4};\;c=0.05;\;h=0.01;\;\zeta =0.05;\;\epsilon =10^{-4};\;\bar{\rho }=1.1;\;\underline{\rho }=-1.1$.

##### Q-Learning

**Figure 2:**
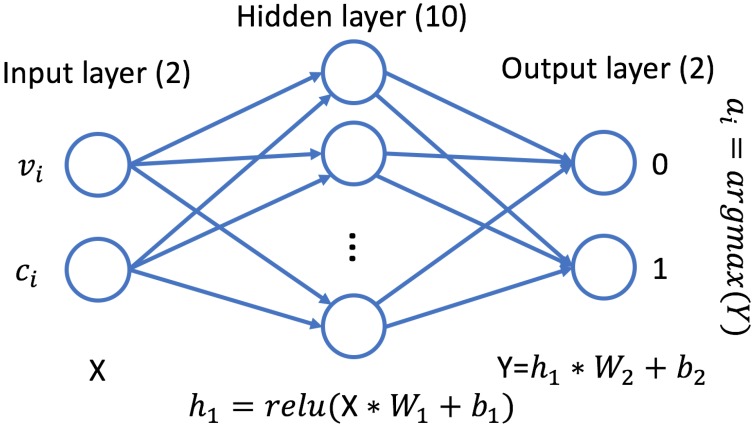
Diagram of the neural network on which the Q-Learning mechanism is based. The inputs are the current value and cost of contribution and the outputs are the weights given to the possible actions. The chosen action is determined by applying argmax to the output of the network.

Q-Learning is a model-free unsupervised reinforcement learning approach that considers both the history and the current context in the decision [29]. The reward in our implementation is defined as $R_{q}=S\left(q,\tau \right)-c_{i}\times a_{i}$, i.e., a component related to the success of the public good, driven by the collective action, and a negative component driven by the individual cost and individual action. The implementation of Q-Learning relies on TensorFlow, the schema of the network is detailed in Figure 2. Q-Learning is influenced by the choice of parameters α, the learning rate, and γ, the learning discount. In our simulations, γ is set to 0, as the future states are independent from the choice of action: the scenario can be classified as contextual bandits, where the reward of an action depends on the current state, but the chosen action does not reflect the next state. A parameter sweep on the value of α showed no impact of the learning rate on the performance of the algorithm; hence, the default value of α=0.001 has been chosen for the experiments.

A disadvantage of reinforcement learning is its sensitivity to initial conditions—for example, a multi-agent learning process might converge to an inefficient equilibrium where nobody contributes. In order to make this outcome less likely, agents are pre-trained to prefer contribution in order to bias the initial exploration period. Pre-training is a reasonable solution as it can be performed during device manufacturing and its effect on the behavior of agents fades off quickly as agents start learning.

### 3.2. Measures

The performance of different contribution strategies is quantified with the following measures (see Table 3):

(a) Success rate: The fraction of the threshold that has been covered by contributions, or 1 if the total contribution is higher than the threshold.
(b) Efficiency: The ratio between the requirement and the sum of contributions. Efficiency is 1 if the sum of contributions is equivalent to the threshold, e.g., all agents contribute 1, it is lower than 1 if the sum of contributions is larger than the threshold.
(c) Social welfare: The average reward, sum of a constant benefit and a constant negative cost (only for contributors).
(d) Privacy: the fraction of agents that did not disclose private information during the current timestep.
(e) Fairness: The Gini coefficient represents the fairness of the current round of contributions (0 is total equality). It is computed for each timestep, as the Gini coefficient of the values that agents contributed in that timestep, and aggregated across time and repetitions.
(f) Fairness over time: It represents the fairness of the history of contributions. It measures the Gini coefficient computed across agents on the individual histories of contribution, from the start of the simulation to the current timestep.

The definition of privacy can be made scenario-specific by adopting an appropriate privacy measure, e.g., K-anonymity, differential privacy [30].

### 3.3. Model Design

This section describes the design of the components in the simulation framework and their interaction.

#### Data Generation

The data generation function contains the logic to generate a new data point for each user and to compute the respective contribution values, contribution costs and common good threshold. The generation function depends on the scenario that is modeled, and might either rely on existing data or generate artificial data randomly. This function is called at the beginning of each timestep in order to provide a new value and cost to each agent, as well as system-wide parameters such as the common good threshold.

#### Decision Function

The decision function maps an input, reflecting the current state of the system, to one of many available actions. The decision function can consider past experience, current context or answer reactively to the current input, depending on the algorithm that is implemented. Some algorithms can improve their performance by learning from feedback about the effect of the action on the environment.

#### Evaluation Function

The evaluation function classifies the current state of the system with respect to a number of measures, which are used to evaluate the behavior of the system. It does so by accessing the state of the system and of all individual agents; this global knowledge of the system allows it to compare the performance obtained empirically with the maximum theoretical performance.

#### Reward Function

The reward function evaluates the effect that the action of an individual agent had on the state of the system and computes a proportionate reward, which might be used to condition the future action of that agent such that they align to some global goal.

#### Supervisor

The supervisor is the main component of the model, which is in charge of coordinating each timestep by updating the state of the system and evaluating the outcome of the set of actions being selected by the population. The supervisor implements the communication system for agents, on which measurements, actions and feedback are exchanged. Another important task of the supervisor is that of evaluating and logging the state of the system for successive analysis. In case of a centralized configuration, the supervisor is also in charge to instruct agents on the actions they have to perform.

#### Agent

The agent class implements generic functions that are required for the working of the simulation environment. The agent class is modular as it can support different logic of operation, e.g., different ways of generating data and different algorithms for decision-making. Agents obtain a new set of input values at the beginning of each timestep, from the respective data generation function. The agent’s own decision function selects one of the available actions for execution, based on the current input and the state of the system: in case of a localized configuration, the agents choose autonomously what action to perform, given the current input values and, possibly, the past experience of the agent. Experience is updated and accumulated by the feedback obtained from the Supervisor. In case of a centralized configuration, actions are decided at the central level and executed by the local decision function.

### 3.4. Parameters of the Model

Table 4 presents an overview of the parameters of the simulation environment. Parameters that have been keep constant across experiments are represented by a number, while parameters that varied across or within experiments, e.g., parameter sweep, are represented as tuples.

## 4. Results and Analysis

This section presents the results of computational experiments. Trade-offs are evaluated between the three contribution strategies presented in Section 3.1 and two baselines: “full” where all users contribute, and “random” where users have 50% probability of contributing at each round (50% chance of contribution does not imply 50% chance of success because each contribution is on average greater than 1.).

All experiments are performed in a Python simulation framework and run on the computing cluster of ETH Zü. Results presented in this paper represent the average state of 20 simulations after 5000 timesteps, and error bars represent the confidence intervals at 95%. The choice of cost, value and public good values is described in Section 2.1.

**Figure 3:**
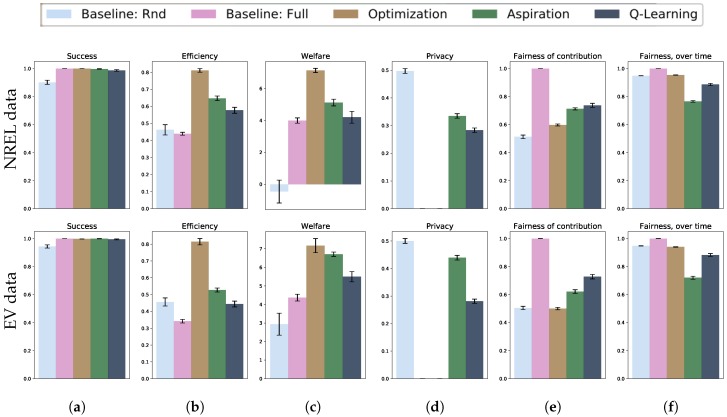
Comparison of contribution strategies. The plots show trade-offs between centralized optimization, aspiration learning and Q-Learning in terms of success rate (**a**), efficiency (**b**), social welfare (**c**), privacy (**d**) and fairness (**e**,**f**). Optimization offers the highest success rate and efficiency, while aspiration learning and Q-Learning offer higher privacy. Q-Learning offers higher fairness than aspiration learning on both measures.

**Figure A1:**
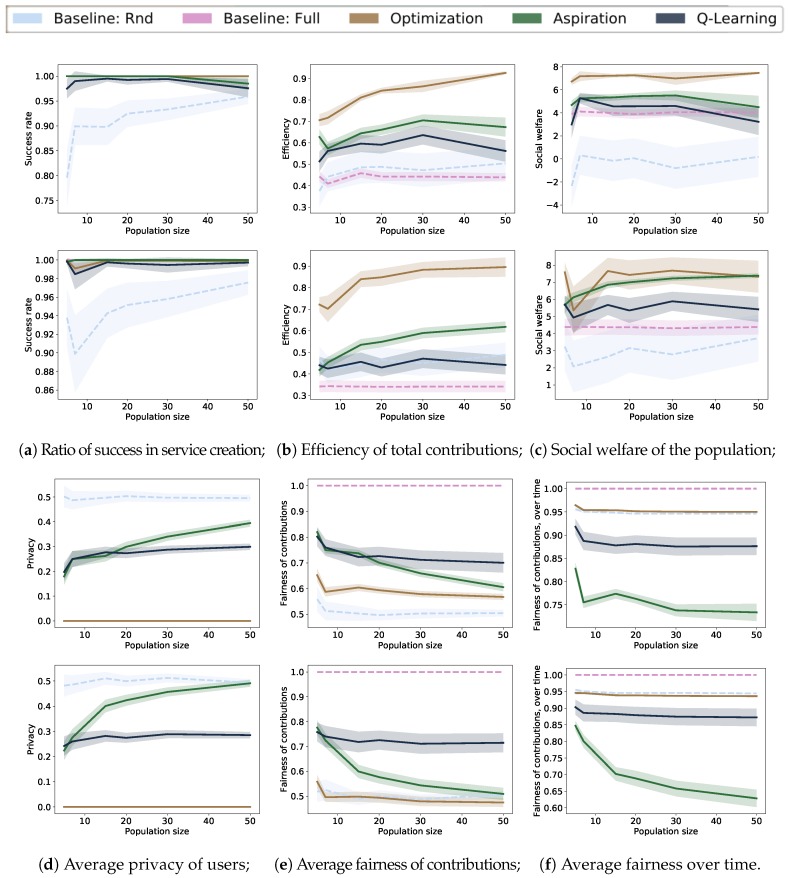
Dependence of results on the population size (*x*-axis). Dashed lines represent baselines, solid lines represent contribution strategies. The plots show that most measures are independent of the population size. This result is driven by the assumption that the public good threshold is proportional to the size of the population. If this threshold were constant, successfully generating the public good would become easier with an increase in the number of agents/contributors. Aspiration learning is the algorithm with the highest dependency on population size, as a larger population size leads to higher efficiency and privacy, as well as lower fairness. This result can depend on the lack of contextual information in aspiration learning, in contrast to Q-Learning, which could lead to reduced performance for smaller and less flexible populations.

**Figure A2:**
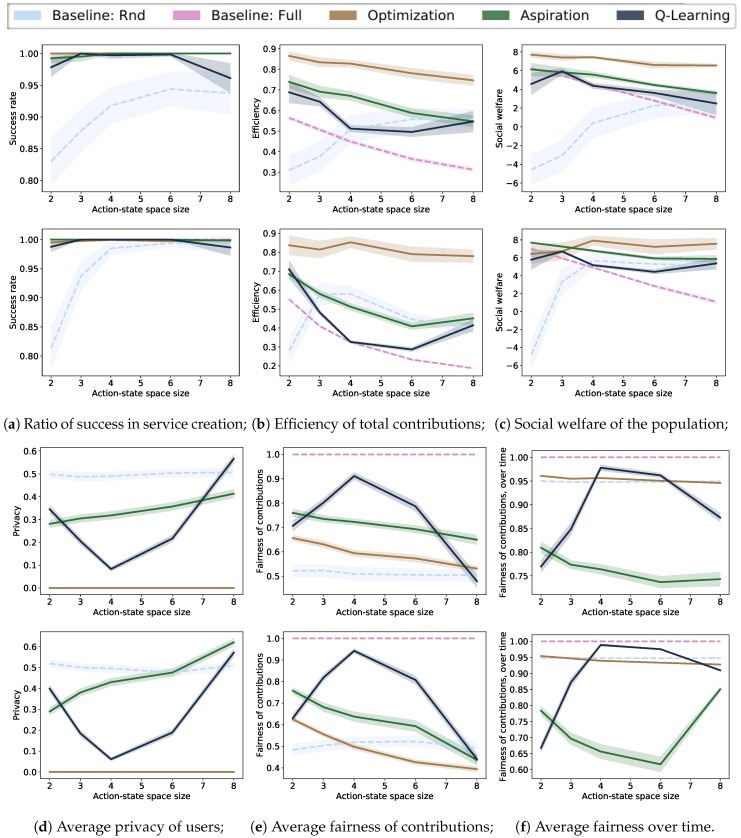
Dependence of results on the range of values and costs (*x*-axis). Dashed lines represent baselines, solid lines represent contribution strategies. The plots show a trend for which all contribution strategies tend to perform worse for larger action-state spaces. Q-Learning is the measure that is most affected by the size of the action-state, in particular, it can be seen that its performance varies significantly on most measures for intermediate sizes. This result is driven by the fixed capacity of the neural network, which makes it more suitable for a certain degree of complexity in the input; the performance of a Q-Learning contribution strategy could be adapted to a given environment by changing the capacity of the network to a more suitable value.

**Figure A3:**
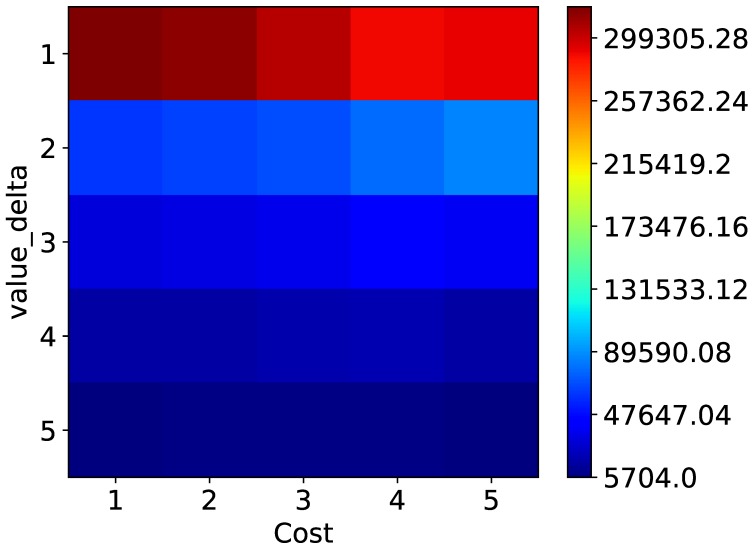
Average frequency of value/cost combinations in the NREL dataset. Data points with a combination of low value and high cost are more frequent in the dataset than data points with high value and high cost. Values are normalized by the maximum speed of the trip and binarized in N integer values.

Full results are shown in Figure 3 and present the comparisons of the contribution strategies by the six measures discussed in Section 3.2. The plots show aggregated results across a range of population sizes, from 5 to 50 users. The decision to aggregate the visualization is motivated by the independence of the results on the size of the population, i.e., the number of sensors, caused by the choice of the public good threshold to be proportional to the size of the population. For completeness, Figure A1 and Figure A2 in the Appendix illustrate the (lack of) variation of these results with respect to the population size and action-state space size. If the threshold would be constant, an increase in the number of sensors, i.e., potential contributors, would make the creation of the public good easier and hence affect all measures.

Success Rate

Concerning success rate (Figure 3a), full contribution and centralized optimization always succeed, i.e., are always able to provide the services, while Q-Learning does not guarantee success and fails in about 2–3% of the cases.

Efficiency

Concerning efficiency (Figure 3b), centralized optimization is the most efficient solution, as it finds the subset of users whose contribution satisfies the requirement at the lowest cost (although the chosen approximation algorithm does not guarantee to find the global minimum). Optimization is not successful if the total requirement is larger than the sum of contributions from all agents, but the experiments are generated to be successful if all users contribute. Efficiency measures how close the total contribution approaches the needs of the system; hence, the baseline in which all agents contribute will have the lowest possible efficiency. Efficiency increases with the size of the population as a higher number of possible solutions—combinations of individual contributions—makes it more likely to find an efficient solution.

Social Welfare

Similarly, optimization scores the highest value of social welfare (see Figure 3c), while localized strategies reach a welfare around 30% lower. Social welfare is the difference between the rewards from the public good and the costs of contributions, so a negative value indicates that costs are higher than gains. Differently from the previous result, the performance of aspiration learning is equivalent to that of Q-Learning, as opposed to that of optimization.

Privacy

Concerning privacy (Figure 3d), neither centralized optimization nor full contribution grant any privacy to the users, as they require full knowledge about the state of the system. Conversely, localized contribution strategies allow a fraction of the user to keep their data private. This fraction increases with the population size for aspiration learning, while Q-Learning trades lower privacy off for higher fairness. Random contribution offers the highest privacy—around 50% of users (as each user has a 50% chance of contributing)—at the expenses of other measures, e.g., success rate and efficiency.

Fairness

Fairness is measured in two ways: “fairness of contributions” compares the actions of all agents at the current time *t* (Figure 3e), while “fairness of contribution over time” considers the histories of contribution up to time *t* (Figure 3f). Full contribution requires all users to contribute; thus, it trades perfect fairness off for other measures such as efficiency. Optimization offers low fairness because it considers only the current state, and users in certain states, e.g., with high values, are more likely to contribute than others.

Fairness over Time

Conversely, optimization offers high fairness over time because agents are randomly assigned to states; hence, the chance of being in any state is over time the same. This result might not hold if states are not randomly assigned, e.g., some users are more likely to obtain high values/costs than others. Aspiration learning bases decisions only on the history of decisions. This leads to higher fairness, as contributions are independent of the state, and to lower fairness over time, caused by individual differences in training that accumulate over time. These values decrease with the population size, while other contribution strategies are not affected by this parameter. Q-Learning scores high values in both measures as it considers both the current context and the history of actions. This allows agents to learn similar behaviors by interacting with one another.

In conclusion, the simulation results highlight a set of trade-offs between the measures of efficiency, privacy and fairness, which are summarized in Table 5. These trade-offs are consistent across scenarios. This suggests that they depend on the characteristics of the algorithms; hence, these results can be the basis for recommending to system designers appropriate contributions strategies for a given cyberphysical system and application domain. Specifically, centralized optimization assures the success of the service and provides high efficiency; it is hence appropriate for mission-critical services for which computation and network constraints are not an issue, e.g., measuring the current load on the smart grid to prevent outages. Localized strategies trade efficiency and reliability off for higher privacy and fairness; therefore, they are best suited for applications where privacy concerns might reduce user adoption, e.g., participatory sensing. Among them, Q-Learning offers the highest fairness; hence, it is ideal for applications where fair access to the service is desirable, e.g., charging of electric vehicles.

## 5. Related Work

The concept of smart city is broad and difficult to define [31]. Many application scenarios can be placed under the umbrella of smart cities [1]. In this work, we focus on a few popular application scenarios: participatory sensing [32,33], with the examples of traffic control [34,35] and traffic congestion maps [36], and smart grid [37], with the example of charging Electric Vehicles (EVs) [38]. The choice of these applications is motivated by the interest shown by the smart city and privacy communities: Privacy is an important component of the smart city [5] that is particularly well studied in the literature, with numerous privacy-preserving solutions being developed for the application scenarios of traffic [39], participatory sensing [20], and the smart grid [40]. Regarding methodology, a variety of solutions have been applied to these application scenarios, ranging from optimization [41,42] to machine learning [43,44].

This work quantifies trade-offs between privacy and fairness. Privacy is a well-studied topic in smart grids [6,45,46] and in participatory sensing [20,47,48], with location privacy being of special interest [49,50,51]. Fairness has been investigated in demand response programs of smart grids [52,53] and in participatory sensing [54], and only recently fairness has been studied independently of the application scenario [7,55,56]. However, only limited literature considers trade-offs between fairness and privacy [57].

These application scenarios have some similarities, e.g., routing protocols are used in both smart grids and traffic control [58]. The most relevant similarity between application scenarios is that both deal with producing a service from user-contributed data. This makes them suitable to be modeled by a Voluntary Contribution Game (VCG) [8], which has been successfully applied to smart grids [12].

In our modeling, we assume that a demand-response algorithm which optimized the use of available energy is in place [59]. This allows us to abstract away details about the smart grid network and focus on individual production and consumption. We also require as the demand-side management algorithm to be privacy preserving, e.g., [60,61]; otherwise, successive privacy considerations would not be meaningful. We have the same requirement for a traffic control algorithm [35] that is privacy preserving [62].

This work analyzes different centralized and decentralized control algorithms. Centralized algorithms are preferable over decentralized ones when it comes to accuracy, as they can take decisions considering the global state of the system. Nevertheless, there are drawbacks that make decentralized algorithm preferable in some particular conditions. The most obvious limitation for centralized algorithms is the intractability of nondeterministic polinomial time (NP) complete problems, e.g., the charging-scheduling problem [63]. Another limitation relates to the communication footprint, which is especially a problem for large wireless sensor networks, as the traffic coming from numerous sensors might saturate the communication medium [64]. A more appropriate solution for this scenario is a decentralized approach that relies on short range communication, or localized algorithms that allow no intra-agent communication and therefore have constant communication footprint. Another advantage of a distributed/localized algorithm over centralized optimization is the increased fault tolerance due to the absence of a single point of failure, i.e., the central optimizer, as well as an increased scalability to large population sizes. Other non-technical constraints might play a role in making decentralized more desirable, e.g., privacy [4] or fairness [53]. Finally, localized algorithms empower the users to choose autonomously about their own actions and to keep ownership of data. A more in-depth comparison of centralized and decentralized algorithms is provided in [26].

Reinforcement learning (RL) algorithms are frequently used in situations where both traditional AI-based approaches, such as planning, or supervising learning are not practical or scalable [65], and where a model of the environment is costly, or even impossible, to obtain. RL has been applied extensively to both smart city applications under consideration, e.g., Ref. [66] uses RL to reduce energy costs, and [67] uses RL in participatory sensing. The main advantage of using RL over other decentralized optimization approaches, e.g., evolutionary computing or Monte Carlo tree search, as evaluated in [26], is that it operates by selecting an action based only on the current state, i.e., current set of conditions, rather than pre-calculating longer-term (e.g., daily) schedules/actions; therefore, it does not need a prediction of the future conditions, and it does not need to re-calculate the schedules if underlying conditions change. Another advantage is that a learning algorithm obtains rewards by interacting with the environment, so it can work in environments in which the relation between state, actions and rewards is not known a priori—for example, because it depends on the collective action of a population. In addition, as Q-learning is a widely established technique, numerous extensions exist that enable its implementation in numerous varieties based on domain requirements, e.g., multi-goal implementations using W-Learning [43,44] and collaborative implementations using Distributed W-Learning [68].

Table 6 compares the current work with the most relevant literature along three dimensions: the kind of target measure, the organization of the system and the application scenario. The table highlights the uniqueness of the current work, as no other work generalizes over application scenarios, while considering trade-offs between privacy and fairness, as well as trade-offs between different system organizations, which is the main contribution of this paper.

## 6. Conclusions and Future Work

The goal of this paper is to provide a tool for understanding user participation in established smart city services. In order to do this, we first proposed a scenario-independent design principle based on voluntary user contribution and a simulation framework for smart city applications. The applicability of this framework was verified using real-world data from the two application scenarios of traffic congestion monitoring and electric vehicle charging. Secondly, we used this framework to measure the effects, along various dimensions, of citizens’ participation.

Voluntary contributions empower users to control the ownership of their resource, e.g., by contributing data towards a service, independently of the type of resource and its use. Different contribution strategies produce trade-offs along measures such as efficiency, privacy and fairness, which we quantified in both implemented scenarios.

Results suggest that such trade-offs depend on characteristics of the algorithms and not on characteristics of the scenarios. Therefore, they can be used as implementation recommendations to service providers and system designers about the choice of contribution strategies, regardless of the specific smart city domain. Specifically, centralized optimization is found to offer the highest efficiency of contributions and to guarantee the success of the application. It is hence recommendable for mission-critical services that require high availability, e.g., load management on a smart grid. Localized strategies are, on the other hand, less efficient and might cause the system to fail with low probability, but compensate for this by improving on privacy and fairness of contribution. Localized strategies are hence more suitable for privacy-sensitive applications that require user adoption, such as participatory sensing, or applications where fairness in redistribution is crucial, e.g., charging of electric vehicles.

Modeling of scenarios with different cost and value characteristics is left to future work—for example, “negative” contribution in traffic congestion, where users contribute to the public good by choosing a longer route over the shortest but congested route [69]. Incentive mechanisms help increase user participation [70] but require quantifying privacy [24], which could also be addressed in future work. The current work considers only localized algorithms, i.e., it assumed no communication between user devices. Relaxing this limitation is a worthy avenue for future work. Communication between user devices would introduce privacy concerns due to data exchange but would allow analysis of new classes of algorithms, such as decentralized optimization. Finally, it would be beneficial to verify the proposed design principle based on public good theory in other smart city domains, in order to further evaluate its generality and suggest as a complete blueprint for smart city applications. Other potential application areas could, for example, include pollution or noise monitoring and real-time public transport information, or any other similar data-rich application relying on user contributions in which system performance, fairness and user privacy are of concern.

## Figures and Tables

**Table 1:** Mathematical notation, in order of appearance.

Math Symbol	Description
	**Time-Independent Variables**
*S*	The service provider
V={1,…,n}	The set of *n* users
$T\in N_{>0}$	The number of rounds in the simulation
*Q*	The total quality of service after *T* rounds
A={D,C}	The action set
	**Functions**
f:V→R	The quality function
S:R×R→R	The success function
G:R×R→R	The payoff function for successful rounds
B:R×R→R	The payoff function for unsuccessful rounds
	**Time-Dependent Variables**
$r_{i}$	The resource produced by user *i*
$v_{i}\in R$	The value associated to disclosing $r_{i}$
$c_{i}\in R$	The cost associated to transmitting $r_{i}$
$p_{i}\in R$	The privacy leaked when disclosing $r_{i}$
$a_{i}\in A$	The action of user *i*
$A_{+}\subseteq V$	The set of contributors
q∈R	The service quality
τ∈R	The quality requirement
G∈R	The payoff for a successful round
B∈R	The payoff for an unsuccessful round
$U_{i}$	The utility of user *i*
	**Scenario: Smart Grid**
$\pi_{i}\in R$	The energy production of household *i*
$\beta_{i}\in R$	The baseline consumption of household *i*
σ∈R	The energy surplus

**Table 2:** Definition of utilities for agent *i*. Let $q_{-i}=\sum_{j\in A_{+}\backslash \left\{i\right\}}v_{j}=q-v_{i}$ be the total contributions, excluding agent *i*, and τ be the global requirement. This game qualifies as a threshold public goods game if $G\left(\tau ,q\right)+B\left(\tau ,q\right)>c_{i}$, which is always verified for large enough values of *G* or *B*.

$q_{-i}$	$q_{-i}+v_{i}<\tau$	$\tau -v_{i}\leq q_{-i}<\tau$	$\tau \leq q_{-i}$
Outcome	Failure	Depends on $a_{i}$	Success
$U_{i}\left(a_{i}=C\right)$	$-B\left(\tau^{t},q^{t}\right)-c_{i}^{t}$	$G\left(\tau^{t},q^{t}\right)-c_{i}^{t}$	$G\left(\tau^{t},q^{t}\right)-c_{i}^{t}$
$U_{i}\left(a_{i}=D\right)$	$-B(\tau^{t},q^{t})$	$-B(\tau^{t},q^{t})$	$G(\tau^{t},q^{t})$

**Table 3:** Measures used during evaluation.

	Measure	Definition
(a)	Success	$\Sigma^{t}=\min\left(1,q^{t}/\tau^{t}\right)$
(b)	Efficiency	$E^{t}=\tau^{t}/q^{t}\;\mathrm{if}\;\tau^{t}\leq q^{t}\;\mathrm{else}\;0$
(c)	Social welfare	$W^{t}=\frac{i}{T}\sum_{t=1}^{T}U_{i}^{t}$
(d)	Privacy	$P^{t}=1-\left|A_{+}^{t}\right|/n$
(e)	Fairness	$F^{t}=\frac{1}{n}\left(n+1-2\left(\frac{\sum_{i=1}^{n}\left(n+1-i\right)y_{i}}{\sum_{i=1}^{n}y_{i}}\right)\right)\;where\;y_{i}=v_{i}^{t}$
(f)	Fairness, over time	$G=\frac{1}{n}\left(n+1-2\left(\frac{\sum_{i=1}^{n}\left(n+1-i\right)y_{i}}{\sum_{i=1}^{n}y_{i}}\right)\right)\;where\;y_{i}=\sum_{t=1}^{T}v_{i}^{t}$

**Table 4:** List of parameters in the simulation environment. Tuples represent the values of parameters that have been tested during the parameter sweep.

Parameter	Values
Simulation length	5000
Repetitions	20
Population size	(5,7,15,20,30,50)
**Scenario: Smart grid**	
Minimum value	1
Maximum value	(2,3,4,6,8,10)
**Scenario: Participatory sensing**	
Minimum value	1
Maximum value	(3,4,6,8,10)
Public Good Threshold	0.8
**Q-Learning**	
Alpha	0.001
Gamma	0.0
Dropout probability	0.8
Hidden layer size	10
**Aspiration Learning**	
λ	$10^{-4}$
*c*	0.05
*h*	0.01
ζ	0.05
ϵ	$10^{-4}$
$\bar{\rho }$	1.1
$\underline{\rho }$	−1.1

**Table 5:** Summary of advantages and disadvantages of contribution strategies.

Type	Algorithm	Pros	Cons
Baseline	Full	Success	Efficiency
Baseline	Random	Privacy	Success
Centralized	Knapsack	Success, Efficiency	Privacy, Fairness
Localized	Aspiration	Privacy	Fairness over time
Localized	Q-Learning	Fairness (over time)	Efficiency

**Table 6:** Comparison of related literature.

	Measure		Organization		Application Scenario		
**Paper**	**Privacy**	**Fairness**	**Centralized**	**Decentralized**	**Smart Grid**	**Traffic Management**	**Participatory Sensing**
Current work	✓	✓	✓	✓	✓	✓	✓
[39]	✓	✗	✗	✓	✗	✓	✗
[67]	✓	✗	✗	✓	✗	✗	✓
[62]	✓	✗	✓	✗	✗	✓	✗
[6,40,45,46,61]	✓	✗	✓	✓	✓	✗	✗
[20,47,48,49,50,51]	✓	✗	✓	✓	✗	✗	✓
[52,53]	✗	✓	✓	✗	✓	✗	✗
[54]	✗	✓	✓	✗	✗	✗	✓
[55]	✓	✓	✗	✓	✗	✗	✓
[7]	✗	✓	✗	✓	✗	✓	✓
[56]	✗	✓	✓	✓	✓	✓	✓
[57]	✓	✓	✓	✗	✓	✗	✗
[4]	✓	✗	✗	✓	✓	✓	✓
